# A High−Performance Anti−Corrosive Epoxy Coating Based on Ultra−Thin Hydroxyapatite Nanosheets with pH−Responsive Functions

**DOI:** 10.3390/molecules28176223

**Published:** 2023-08-24

**Authors:** Chun Feng, Lijuan Zhu, Legang Chen, Xuezhi Hui, Jinling Liu, Lei He, Xiaofeng Bai, Zongxue Yu

**Affiliations:** 1 Tubular Goods Research Institute, China National Petroleum Corporation, Xi’an 710077, China; fengchun003@cnpc.com.cn (C.F.); 202121000248@stu.swpu.edu.cn (L.H.); baixiaofeng@cnpc.com.cn (X.B.); 2 State Key Laboratory for Performance and Structure Safety of Petroleum Tubular Goods and Equipment Materials, Xi’an 710077, China; 3 School of Chemistry and Chemical Engineering, Southwest Petroleum University, Chengdu 610500, China; 202122000288@stu.swpu.edu.cn; 4 Petrochina Changqing Oilfield Company, China National Petroleum Corporation, Xi’an 710021, China; huxz7_cq@petrochina.com.cn; 5 Bureau of Geophysical Prospecting Inc., China National Petroleum Corporation, Zhuozhou 072751, China; liujinling03@cnpc.com.cn

**Keywords:** hydroxyapatite, multifunctional anticorrosion, intelligent coating

## Abstract

The changes in the working environment have necessitated greater requirements in terms of the long−term anti−corrosion ability of metal anti−corrosion coatings, and the emergence of intelligent coatings has met this demand. A nanocontainer with a hydrophobic inner cavity and hydrophilic outer cavity called β−cyclodextrin (β−CD) was grafted onto the surface of hydroxyapatite (HAp) with a silane coupling agent, encapsulating benzotriazole (BTA) and embedded in epoxy resin to improve the coating anticorrosion performance. The excellent corrosion resistance of the coating in immersion and scratch experiments was derived from the inert protective layer formed by the reaction of the rapidly released corrosion inhibitor with the corrosion products on the metal surface. After 30 days of immersion experiment, the coating could still maintain the low−frequency impedance value of 6.28 × 10^7^ Ω cm^2^. In this work, the enhancement of the physical barrier function of HAp nanoparticle and the pH−response function conferred by β−cyclodextrin provided the coating with good passive and active acting abilities in corrosive environments, respectively.

## 1. Introduction

Undeniably, the annual economic losses caused by metal corrosion and the negative impact on the environment are immeasurable. In addition, the safety risks caused by metal corrosion also have to be considered. The current methods of metal protection include synthesis of corrosion−resistant alloys [1], use of corrosion−resistant coatings [2], addition of corrosion inhibitors [3,4,5], cathodic protection and anode passivation [6], etc. It is worth mentioning that epoxy resin anticorrosive coatings are favored in practical applications because of their high efficiency, economic efficiency, and simple operation [7,8,9,10,11]. Epoxy resin is widely used in practical industrial production. For example, the impermeability, high temperature resistance, and corrosion resistance of the graphene−modified coating can effectively resist the corrosion of pipelines in high−temperature and saline environments. Juan Chu et al. improved the coating by adding RGO to the epoxy resin, and the prepared coating showed good corrosion resistance under high−temperature and high−saline test environments [12].

However, the evaporation of the solvent in the curing process of the thermosetting resin will inevitably cause caustic medium to enter the pores on the coating surface, which is a problem that has to be solved, otherwise the performance of the coating will be greatly reduced. Coatings that can repair themselves when corrosion occurs, thus extending the protective life, are called “smart−coatings” [13,14,15]. Actually, “smart−coatings” have been reported in recent studies. Nanomaterials with cavity structures are used as containers to load active corrosion inhibitors that provide the coating a self−healing function and add them to the epoxy coating to prepare the “smart−coating”, and mesoporous silica (SiO_2_) [16,17,18], alloxite nanotubes (HNT) [19,20,21], carbon nanotubes (CNT) [7,22,23], and metal−organic frames (MOFs) [5,24,25,26] are all excellent nanocontainers.

Cyclodextrins (CDs) [27] are produced by the glucuronic acid unit at the 1,4−glucoside bond and have the potential to be nanocarriers due to their annular structure and internal cavities where corrosion inhibitors can be loaded. The different types of cyclodextrins depend on the number of glucopyranose units. For example, α−cyclodextrin, β−cyclodextrin, and γ−cyclodextrin consist of 6, 7, and 8 glucopyranose units, respectively [28,29]. Meanwhile, β−cyclodextrin (β−CD) has a larger cavity size compared with the other two cyclodextrins, and the R−OH and OH functional groups in β−CD form a hydrophilic outer cavity and a hydrophobic inner cavity, which provide conditions for better load corrosion inhibition [30,31].

Unfortunately, the continuous improvement of anti−corrosion coating performances is due to the metal’s progressively more harsh environment, which means that only a single functional anticorrosive coating is insufficient. Epoxy coatings with both active and passive anticorrosive properties have therefore been reported.

Hydroxyapatite (HAp) is a natural mineral with the chemical formula Ca_10_(PO_4_)_6_(OH)_2_ and is a major component of animal teeth and bones. HAp has been widely used in various fields in recent years due to its high crystallinity, easy modification, adjustable form, and high biocompatibility [32,33,34,35]. Hydroxyapatite is considered to be an ideal candidate material for hard tissue repair. For example, the synthetic HAp grafts reported by Zhou et al. [34]. combined well with host bone to promote the formation of new bone, and could also combine HAp with new bone to promote the recovery of damaged bone tissue. Subsequent studies in rabbits showed that the graft had advantages in binding to host bone as well as induction and integration with new bone compared to allografts and bone grafts with low HAp content. Okabayashi et al. [36] activated HAp fibroblasts and accumulated vascular endothelial cells to support skin wound healing, and the HAp bioceramics were in close contact with skin tissue and adhered firmly to prevent bacterial infection at exit sites and tunnels. Moreover, HAp is a new environmentally friendly adsorbent for removing toxic heavy metals due to its high surface area, high stability, low water solubility, and excellent ion exchange properties. Saber−Samandari et al. [37,38] successfully prepared chitosan/Fe−HAp nanocomposites and effectively removed the heavy metal lead. The application of coatings to medical metal implants is a hot topic in the medical field because few materials are capable of both high biocompatibility and corrosion. Chew et al. [39]. showed that HAp coatings prepared by electrophoretic deposition could improve the corrosion resistance of stainless steel in a simulated human environment (SBS) resistance model. Xue et al. [40]. prepared a structurally stable GO−HAp nanocomposite via an in−situ growth technique. The results showed that the impedance value of the Go−hap/EP coating was 7 times that of pure EP coating after a 72 h electrochemical impedance spectroscopy test. This is because the synergistic action of GO and hydroxyapatite enhances the anti−corrosive properties of the epoxy resin coating.

In this paper, a composite epoxy coating with passive and active anticorrosive functions conferred by the addition of composite material BTA−HAp−CD was prepared. First, HAp−CD was prepared by loading β−CD onto the HAp surface under the action of silane coupling agent KH560, and then the corrosion inhibitor BTA was encapsulated in the cavity structure of β−CD by the vacuuming method; the resulting product was named BTA−HAp−CD. FTIR, XRD, XPS, and SEM were used to characterize the composite materials, and then the intelligent release of the nanocontainer under different pH conditions was detected by UV−vis spectral release experiments. Finally, XPS and EDS detection methods were used to verify the successful loading of BTA. In addition, we also studied the dispersion properties of different composites in different solvents and epoxy resins. Finally, the anti−corrosive properties of composite coatings with different materials were evaluated by electrochemical corrosion experiments (EIS) and artificial scratch self−healing experiments. The composite material synthesized in this paper was not only simple in terms of its fabrication method, but also cheap, and did not display a weaker corrosion resistance than other two−dimensional fillers; therefore, it has broad application prospects in the field of anti−corrosive metal coatings.

## 2. Results and Discussion

### 2.1. Characterization of HAp, F−HAp, β−CD, HAp−CD, and BTA−HAp−CD

**Figure 1:**
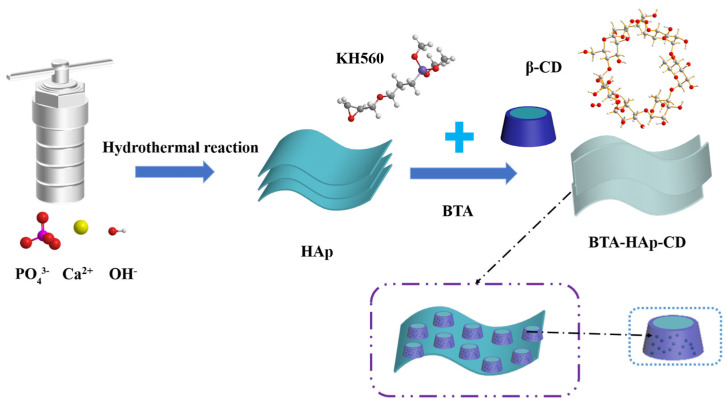
Flow chart of the synthesis of β−CD grafted on Hap’s surface and loaded with corrosion inhibitor BTA.

**Figure 2:**
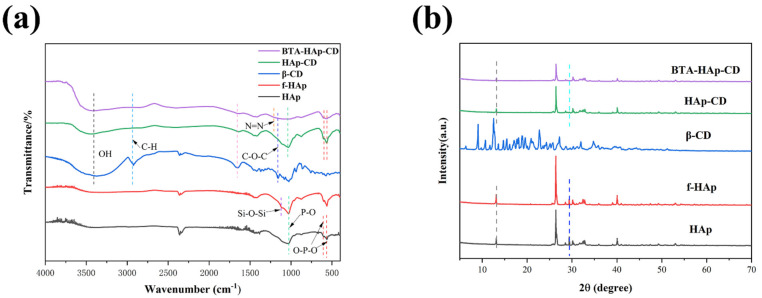
FT−IR (**a**) and XRD (**b**) patterns of the different materials.

Flow chart of the synthesis of BTA−HAp−CD are shown in Figure 1. Figure 2a shows the infrared spectra of HAp, F−HAp, β−CD, HAp−CD, and BTA−HAp−CD. The vibration peak at 1029 cm^−1^ is derived from the asymmetric stretching of the phosphate group’s P−O bond in the HAp spectrogram. Meanwhile, vibration peaks appeared at 601 cm^−1^ and 564 cm^−1^ due to the bending vibration of the O−P−O bond in the phosphate group. After comparison, it was found that the spectra of F−HAp were consistent with HAp except for the vibration peak at 1124 cm^−1^ due to the stretching vibration of the Si−O−Si bond, which indicated that the modification of the silane coupling agent KH560 was successful. For β−CD, the peaks in the spectrum are derived from stretching vibrations of OH at 3411 cm^−1^, C−H at 2934 cm^−1^, and C−O−C at 1161 cm^−1^. The spectrum of composite material BTA−HAp−CD contains characteristic peaks of all monomer materials. The new absorption peak at 1211 cm^−1^ is derived from N=N in the corrosion inhibitor BTA [41], which preliminarily indicates that composite material A was successfully synthesized.

X−ray diffraction patterns of different materials are shown in Figure 2b. The crystal characteristics of the composite were determined by comparing the diffraction pattern of the prepared sample with the standard. The characteristic peaks appeared at 2θ = 13.13°, 26.3°, 29.4°, and 40.1° in the HAp diffraction pattern, which are consistent with the standard characteristic peaks [9]. It can be seen from the diffraction pattern of F−HAp that the modification of the silane coupling agent did not change the crystal morphology of HAp as its pattern was consistent with Hap [42]. For HAp−CD, the diffraction pattern was almost the same as HAP, but it was still found that the height of the diffraction peak decreased, indicating that the modification of 1 had an effect on the crystallinity of HAP but did not change the location of the diffraction peak. Loading BTA in β−CD did not change the diffraction pattern; therefore, the patterns of BTA−HAp−CD and HAp−CD were consistent.

**Figure 3:**
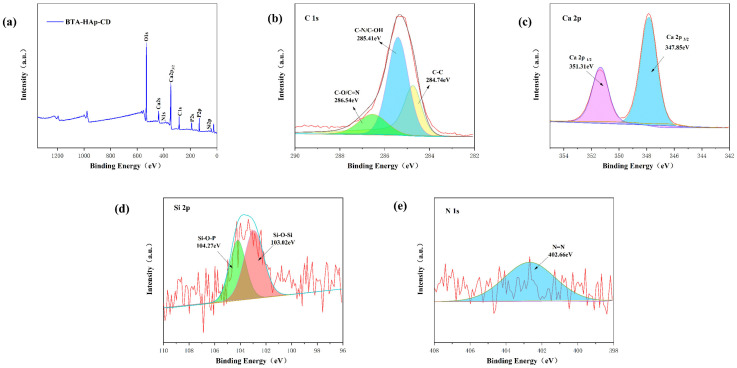
XPS of BTA−HAp−CD: full spectrum (**a**) and fine spectrum (**b**−**e**).

X−ray photoelectron spectroscopy analysis of the material’s elemental composition is required to determine whether the composite has been successfully prepared. Figure 3a shows the full spectrum of XPS analysis, and it can be seen that C, N, Ca, O, P, and Si elements were contained. The fine spectrum of C (Figure 3b) showed three fitting peaks with binding energies of 284.74 eV, 285.41 eV, and 286.54 eV, respectively, whose corresponding valence bonds were C−C, C−N, and C−O/C=N, respectively. Moreover, the two fitting peaks of binding energy 351.31 eV and 347.85 eV shown in the fine spectrum of Ca 2p are derived from Ca 2p_1/2_ and Ca 2p_3/2_, respectively [43]. Figure 3d shows the fine spectrum of Si 2p, and it can be seen that the Si−O−Si bond had a binding energy of 103.02 eV and the Si−O−P bond had a binding energy of 104.27 eV. The fitting peak of binding energy 402.66 eV shown in the fine spectrum of N 1s represents N=N, which is derived from the corrosion inhibitor BTA, so it can be inferred that the corrosion inhibitor loading was successful [44]. Therefore, the successful preparation of the composite material BTA−HAp−CD can be confirmed by XRD, FT−IR, and XPS analysis.

Figure S1 shows the comparison of the stability of different materials dispersed in different solvents at different times. From left to right, the bottles were HAp, F−HAp, HAp−CD, and BTA−HAp−CD. All four materials remained fairly dispersible after 2 h of standing in deionized water, but partial precipitation occurred at the bottom of bottles containing HAp when the standing time reached 12 h. However, after 24 h of standing, all the materials in the bottles except the bottle containing BTA−HAp−CD completely precipitated, as shown in Figure S1a–c. Similarly, only BTA−HAp−CD maintained good dispersion after 24 h of standing experiment with anhydrous ethanol as the solvent, as shown in Figure S1d–f, which means the modification of the material improves its dispersion in solvents and may also greatly improve its dispersion in epoxy resins.

**Figure 4:**
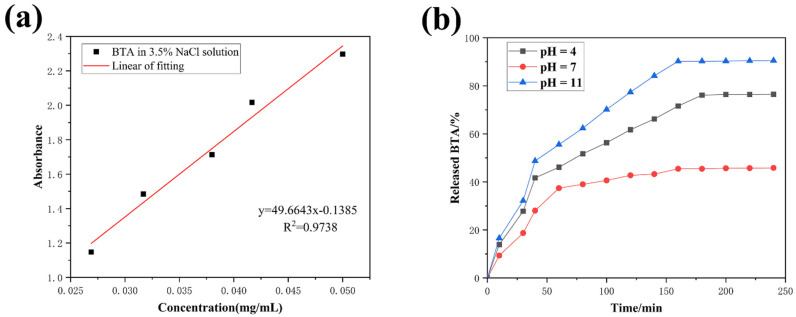
BTA standard release curve (**a**) and release experiment at different pH values (**b**).

The release behavior of BTA was systematically tested in buffer solutions of different pH values. The results showed that the release of BTA was dependent on pH and time. First of all, it can be observed from Figure 4 that regardless of the pH media, the release curve showed a faster release rate in the initial stage, which would enable the coating to have a faster reaction rate when corrosion behavior occurs suddenly, thus minimizing the damage to the coating. Moreover, it can be seen from the release curve that acidic and alkaline media were more conducive to the release of BTA, which is attributed to the collapse of β−cyclodextrin molecular load structure in acidic and alkaline media. The release rates of corrosion inhibitors reached 76% and 90% in acidic and alkaline media, respectively, while only 45% in neutral media, which demonstrates the ability of the synthesized composite to confer a pH response on the coating.

**Figure 5:**
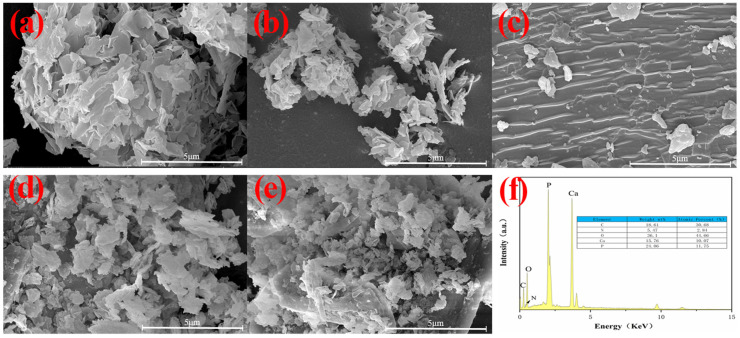
SEM of HAp (**a**), F−HAp (**b**), β−CD (**c**), HAp−CD (**d**), and BTA−HAp−CD (**e**), and the EDS of BTA−HAp−CD (**f**).

Figure 5 shows the microscopic morphology of HAp, F−HAp, HAp−CD, and BTA−HAp−CD under scanning electron microscopy. The lamellar structure of HAp with a smooth surface is observed in Figure 5a, while the surface of F−HAp modified by KH560 only became rougher and still exhibited a lamellar structure that was the same as HAp. In addition, the increase in the thickness and the non−uniformity of the surface roughness of BTA−HAp−CD can be observed compared with HAp, which confirmed the successful preparation of the composite material.

### 2.2. Characterization of the Composite Coatings

**Figure 6:**
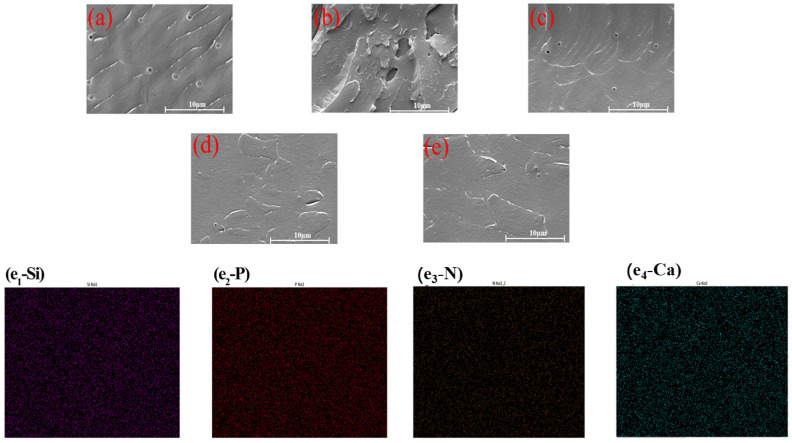
SEM of coating cross−section: pure EP (**a**), HAp/EP (**b**), F−HAp/EP (**c**), HAp−CD/EP (**d**), and BTA−HAp−CD/EP (**e**), and the element detection of BTA−HAp−CD/EP (**e_1_**–**e_4_**).

The dispersion uniformity of the nanofiller in the epoxy resin is an important index to judge whether it can improve the coating’s corrosion resistance. The dispersion of nanocontainers in coatings can be judged by observing the cross−sectional surface of the coatings, as shown in Figure 6 in SEM. It was found that the pure EP coating had a smooth cross−section with few defects other than pores due to solvent evaporation during curing from Figure 6a. A significant agglomeration was observed in the epoxy coating section with HAP added. However, HAP modified by KH560 showed good dispersion in epoxy coating, and the two materials performed completely differently in epoxy resin due to the enhancement of the interface interaction between the two−dimensional nanomaterials and the epoxy collective by KH560. In addition, the cross section of the coating with BTA−HAp and BTA−HAp−CD was smooth and the porosity was significantly reduced due to the heat causing evaporation of the solution. The composite material has good compatibility with epoxy resin according to the distribution of elements in the coating, as exhibited in Figure 6e (Ca, P, N, Ca).

**Figure 7:**
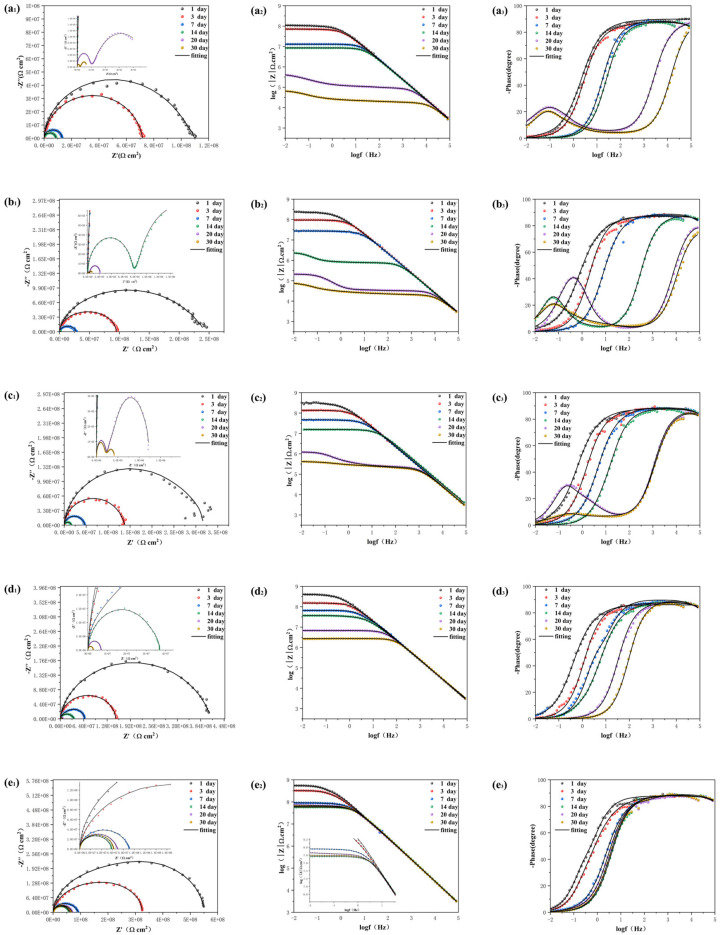
EIS of pure EP (**a_1_**–**a_3_**), HAp/EP (**b_1_**–**b_3_**), F−HAp/EP (**c_1_**–**c_3_**), HAp−CD/EP (**d_1_**–**d_3_**), and BTA−HAp−CD/EP (**e_1_**–**e_3_**) coatings soaked in the 3.5% NaCl solution for different times.

The EIS experiments were conducted by immersing the coating in a 3.5% NaCl solution for various durations to investigate the corrosion resistance of the prepared epoxy coating in real−world conditions. The arc observed in the Nyquist diagram, known as the capacitive reactance arc, can be directly utilized to assess the barrier performance of the coating based on changes in its radius [30,45]. Figure 7 corresponds to the Nyquist diagram, Bode diagram, and phase angle diagram of the pure EP coating (a), HAp/EP (b), F−HAp/EP (c), HAp−CD/EP (d), and BTA−HAp−CD/EP (e) soaked in 3.5% solution for different times, respectively. During the initial 14 days of immersion, the capacitive reactance of pure EP coating, HAp/EP, and F−HAp/EP decreased sharply and two capacitive arcs appeared after 14 days of immersion, which means that defects appeared in the coatings and more than one capacitor loop appeared in the electrochemical system. Moreover, it can be seen from the sharp decline of impedance value in the Bode diagram and the appearance of multiple time constants in the phase angle diagram that corrosion behavior occurred on the metal substrate surface. The rapid degradation of the coating performance is due to the micropores created by solvent evaporation during the curing process, which act as a rapid channel for corrosive media to invade the coating. Although the dispersion of modified HAp in the coating was greatly improved and the corrosion resistance of the coating was also improved according to the electrochemical data, the original micropore defects of the coating were not solved. On the contrary, coatings HAp−CD/EP and BTA−HAp−CD/EP still maintained a reactance arc in the Nyquist diagram and a time constant in the phase angle diagram after soaking in 3.5% NaCl solution for 30 days. Although the impedance in the Bode diagram decreased, both of them were within a range such that the coatings could still maintain good corrosion resistance, which proves that the addition of β−cyclodextrin has a practical effect on improving the long−term corrosion resistance of the coating. Similarly, the role of corrosion inhibitors in the corrosion protection process can also be demonstrated by electrochemical data. For example, the HAp−CD/EP coating maintained a higher level of impedance than the BTA−HAp−CD/EP coating after 30 days of immersion in the Bode diagram, and the Nyquist arc radius of the BTA−HAp−CD/EP coating showed some increase after 14 days of immersion [46].

Generally speaking, low−frequency impedance (|Z|_f = 0.01 Hz_) values in Bode diagrams can be used to evaluate the barrier properties of coatings. The higher the |Z|_f = 0.01 Hz_ value, the better the barrier performance. During the first 14 days of immersion, the |Z|_f = 0.01 Hz_ value dropped rapidly to 8.45 × 10^6^ Ω cm^2^ for the pure EP coating, which was a drop of almost two orders of magnitude [47]. The rapid decline of the |Z|_f = 0.01 Hz_ value is due to the acceleration of the corrosive medium into the coating due to the defects of the pure EP coating itself, but the coating can still play a blocking role. However, it can be seen from the data that the |Z|_f = 0.01 Hz_ value of the coating with HAp added decreased more than that of the pure EP coating after 14 days of immersion because the agglomeration phenomenon caused by the poor interfacial compatibility between HAp nanosheets and epoxy resin matrix caused more serious defects in the coating. Although the |Z|_f = 0.01 Hz_ values of coatings F−HAp/EP, HAp−CD/EP, and BTA−HAp−CD/EP showed a downward trend during the 14 days of immersion experiment, they were still higher than those of pure EP and HAp/EP coatings, and the long−term retention of the coating properties is due to the better interfacial compatibility of the composite with the epoxy resin matrix and the enhanced barrier properties of the coating. In addition, the |Z|_f = 0.01 Hz_ value of the BTA−HAp−CD/EP coating increased to a certain extent when the immersion experiment reached 14 to 16 days, which proved the release of corrosion inhibitor BTA in the coating. After soaking in 3.5%NaCl solution for 30 days, the |Z|_f = 0.01 Hz_ values of pure EP, HAp/EP, F−HAp/EP, and HAp−CD/EP coatings all decreased significantly to only 6.44 × 10^4^ Ω cm^2^, 2.08 × 10^5^ Ω cm^2^, 4.19 × 10^5^ Ω cm^2^, and 2.64 × 10^6^ Ω cm^2^, respectively, which indicated that the epoxy matrix itself completely lost its protective function. We can also draw the same conclusion by analyzing the breakpoint frequency (f_b_), which is the frequency when the phase angle was 45° [48]. The value of the f_b_ is used to reflect the corrosion and delamination of the microscopic region between the interface of epoxy resin and metal substrate, and it is inversely proportional to the protection performance of the coating. The f_b_ of all coatings showed an increasing trend during the immersion experiment because the occurrence of corrosion behavior led to the generation of corrosion products on the interface between the coating and the metal, which intensified the degree of stratification. Obviously, only the BTA−HAp−CD/EP coating reduced the occurrence of this condition due to the action of the corrosion inhibitor, which could keep the f_b_ at a low level during the 30−day immersion experiment, also explaining why only the BTA−HAp−CD/EP coating maintained a |Z|_f = 0.01 Hz_ value close to 1.0 × 10^8^ after a long−time immersion experiment.

**Figure 8:**
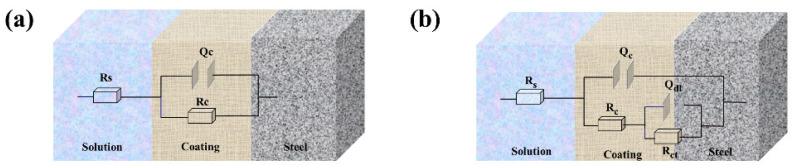
Equivalent circuit diagrams before (**a**) and after (**b**) of the coatings were invaded.

**Figure 9:**
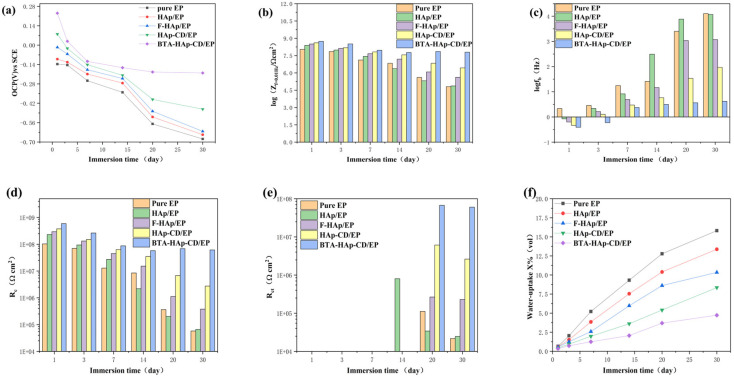
Electrochemical fitting parameters of the coatings: OCP (**a**), log |Z|_f = 0.01 Hz_ (**b**), log f_b_ (**c**), R_c_ (**d**), R_ct_ (**e**), and water absorption rate (%) (**f**).

In order to better study the corrosion behavior of the coating during the soaking process, the equivalent circuit diagram shown in Figure 8 was used to simulate the electrochemical system of the coating. The reason for using two different equivalent circuit diagrams is that the electrochemical behavior of coatings in different time periods is different, and the classification is based on the number of time constants in the phase angle diagram. There was only one time constant in the phase angle diagram when the epoxy resin matrix still played a barrier role, while there were two time constants in the phase angle diagram when the corrosive medium passed through the coating and corroded the metal substrate. The circuit elements R_s_, Q_c_, R_c_, R_ct_, and Q_dl_ in the equivalent circuit represent the electrolyte resistance, coating capacitance, coating resistance, charge transfer resistance, and double layer capacitance, respectively [49,50,51]. Specific data is shown in Table S1. R_c_ and R_ct_ can reflect the changes in the porosity and degradation degrees of the coating during the soaking process, and the specific values obtained by fitting the equivalent circuit diagram model are shown in Figure 9. The R_c_ and R_ct_ of all coatings showed a decreasing trend during the 30−day immersion experiment. However, the impedance of the BTA−HAp−CD/EP coating retained the highest value after 30 days, i.e., one to three orders of magnitude higher than that of the other coatings, demonstrating the best corrosion resistance of all the coatings [4].

The water absorption rate of coating is often the most intuitive index to evaluate the performance of coating in practical application. Water absorption is calculated by the following formula [18,50,52]:

$$X_{v}\%=\frac{\log\left(\frac{C_{c}\left(t\right)}{C_{c}\left(0\right)}\right)}{\log\left(80\right)}\times 100$$

where C_c_(t), C_c_(0), and $X_{v}$% in the equation represent the coating capacitance of the coating substrate at time t, the coating capacitance at the beginning of soaking time, and the water absorption of the coating, respectively. The water absorption rate of the coating will increase with the diffusion of water solution in the coating, and the water solution will invade the coating quickly and accelerate its failure if the water absorption is too high. The water absorption of the pure EP coating reached up to 15.8% after 30 days of immersion, while the water absorption of the BTA−HAp−CD/EP coating was only 4.72%, which confirmed that the addition of BTA−HAp−CD can effectively enhance the corrosion resistance of the epoxy resin. 

**Figure 10:**
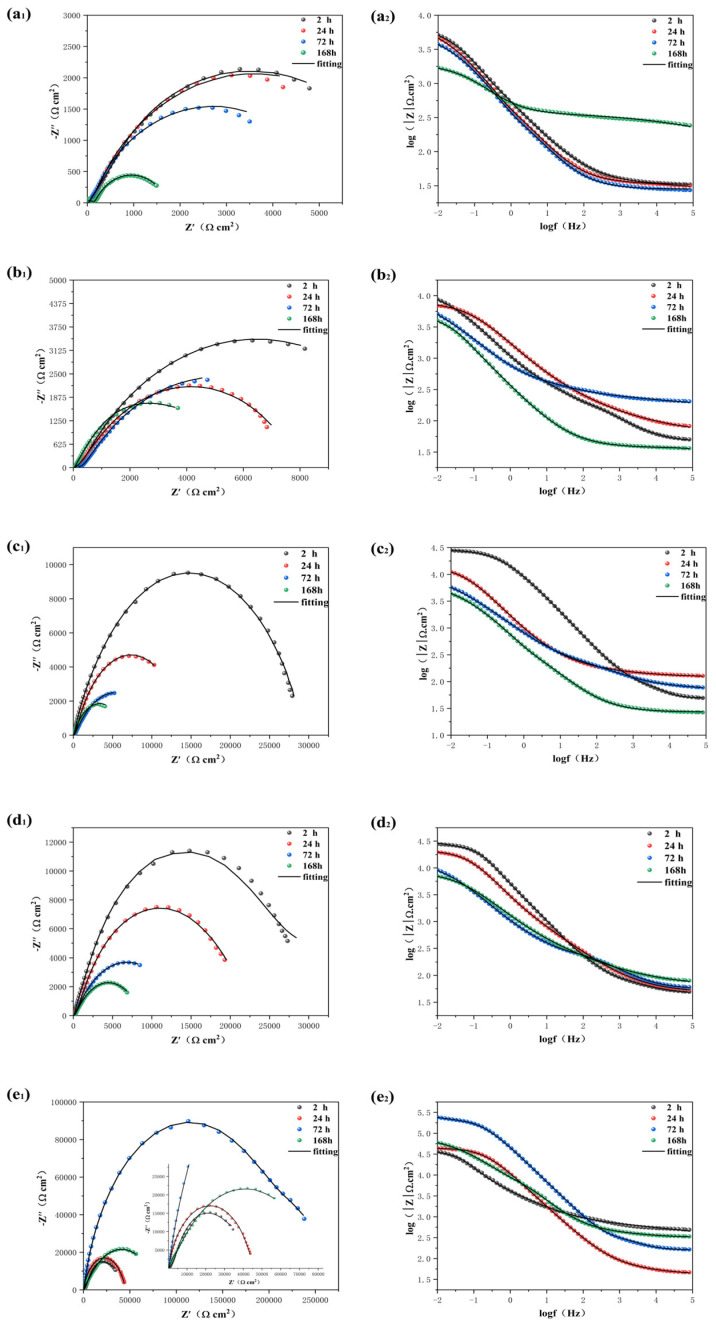
EIS of the scratched coatings: pure EP (**a_1_**,**a_2_**), HAp/EP (**b_1_**,**b_2_**), F−HAp/EP (**c_1_**,**c_2_**), HAp−CD/EP (**d_1_**,**d_2_**), and BTA−HAp−CD/EP (**e_1_**,**e_2_**) soaked in the 3.5% NaCl solution for different times.

**Figure 11:**
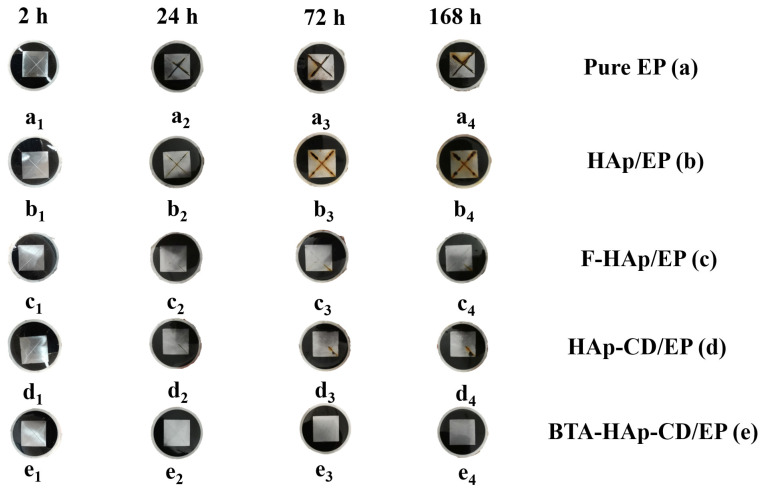
Optical pictures of the coatings after soaking for different times.

In order to show the role of corrosion inhibitors in the coating more directly, the scratch experiment of the coating was carried out. Firstly, we applied artificial scratches on the surfaces of different coatings, and then immersed them in 3.5% NaCl solution and conducted electrochemical tests. The Z_f=0.01Hz_ of scratch test data is shown in Table S2. It can be seen from the electrochemical data in Figure 10 that the resistance of the coating decreased significantly after the manual scratching operation, which is because the corrosive medium can directly corrode the metal surface through the scratching. Moreover, only the BTA−HAp−CD/EP coating impedance value increased at 72 h due to the release of the corrosion inhibitor during the 168 h of immersion experiment, as shown in Figure 11, and the same conclusion can be obtained from the optical picture of the coating as there was no change observed on the surface of the coating due to corrosion [53]. To test the thermal stability of the composite coating, we performed a TGA test on it (Figure S2). It can be seen from the curve that the weight loss of the coating mainly appeared after 400 °C and the weight loss of 100–200 °C was derived from the solvent that was not evaporated in the coating as well as the decomposition of the composite material. Therefore, the epoxy coating prepared in this paper can maintain a high thermal stability within 400 °C.

**Figure 12:**
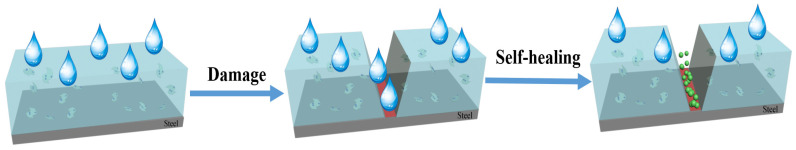
Self−healing mechanism of BTA−HAp−CD/EP coating.

The BTA−HAp−CD/EP coating prepared in this paper is a kind of intelligent coating with both a physical barrier and chemical protection. Its protection mechanism is shown in Figure 12. When the corrosive medium invades, the coating will play its role as a physical barrier. Due to the addition of the BTA−HAp−CD composite material, the porosity inherent on the surface of epoxy resin caused by the evaporation of diluent was greatly reduced, which means that the channel of the corrosive medium into the coating was also greatly reduced. Moreover, the “maze effect” generated by the two−dimensional material extended the length of the channel through which the corrosive medium invaded the coating, thus improving the protective performance of the coating [25,54,55]. When the corrosive medium reached the metal surface and corroded, the nano−container β−CD, which is highly sensitive to pH value, released the corrosion inhibitor BTA stored in the cavity due to the change of pH value of the corroded site. The corrosion inhibitor reacted with the corrosion products to form a passivation protective film covering the metal surface. In addition, the rate of release of corrosion inhibitor changed according to the speed of pH change. Therefore, the intelligent coating of BTA−HAp−CD/EP can realize the effective long−term protection of metal.

## 3. Experimental Section

### 3.1. Materials

Ca(NO_3_) _2_⋅4H_2_O (AR), NH_4_H_2_PO_4_ (AR), NaCl (AR), CH_3_COOH (AR), NaH (AR), β−cyclodextrin (β−CD), KH560, benzotriazole (BTA, AR), acetone (AR), N,N−dimethylformamide (DMF), and anhydrous ethanol (AR) were all purchased from Cologne Reagent Chemical Factory in Chengdu (China). Epoxy resin (WSP−6101) and the curing agent (polyamide) were purchased from Blue Star Technology (Beijing, China) and the N80 steel sheet (1 cm × 1 cm × 3 mm) was purchased from Yubei Yufan Metal material processing plant (Chongqing, China).

### 3.2. Synthesis of Composite Materials

#### 3.2.1. Preparation of HAp

The HAp material was prepared by hydrothermal synthesis. First, 5.9 g Ca(NO_3_)_2_·4H_2_O and 1.72 g NH_4_H_2_PO_4_ were added into a beaker containing 50 mL deionized water, and ultrasonic stirring was performed for 2 h until a uniform dispersion was achieved. Next, 4.5 g urea was taken as the precipitator and added to the uniformly dispersed solution. After half an hour of ultrasonic agitation, the abovementioned solution was transferred to the hydrothermal reactor and kept at 120 °C for 3 h. After the reaction was completed, the reactor was cooled to room temperature, washed with deionized water and ethanol 5 times, respectively, and centrifuged to separate excess impurities. Finally, the solid sediment was dried in a vacuum oven for 12 h.

#### 3.2.2. Preparation of F−HAp

For the preparation of F−HAp, 90 mL ethanol and 10 mL deionized water were measured and placed in a three−way flask, and then 2.5 g KH560 was weighed and added into the solution for full mixing. The pH of the solution was adjusted to 5 by using glacial acetic acid with a glue head dropper. The mixed solution with an adjusted pH value was hydrolyzed by ultrasonic for 30 min and then 1.5 g HAp nanosheets were added. After reaction at 50 °C for 1 h, the reaction was allowed to stand at room temperature for 3 h for centrifugal washing with ethanol and water. Finally, the product was dried in a vacuum oven at 65 °C for 24 h.

#### 3.2.3. Preparation of HAp−CD

We weighed 1.5 g β−cyclodextrin and placed it in a three−way flask containing 150 mL DMF, and then added 0.15 g NaH into the mixture and stirred it magnetically for 5 min at room temperature until no bubbles emerged. Next, the unreacted NaH was removed by filtering. Then 2.5 g F−HAp was added into the filtrate and the round−bottled flask was placed in an oil bath at 110 °C, heated to 110 °C under nitrogen protection, and stirred for 24 h. The resulting solid products were washed in sequence with DMF, methanol, deionized water, and acetone. Finally, the target product was obtained by vacuum drying at 80 °C for 8 h.

#### 3.2.4. Preparation of BTA−HAp−CD

For the preparation of BTA−HAp−CD, 0.5 g of the prepared BTA−HAp−CD material was weighed and placed in a beaker containing 100 mL ethanol for ultrasonic dispersion for 30 min, and then 5 g BTA was added into the abovementioned solution and magnetically stirred at room temperature for 6 h. Thereafter, the suspension was transferred to a distillation flask in a rotary evaporator and vacuumed with a vacuum pump. After the suspension was kept under vacuum for 1 h, it returned to normal atmospheric pressure. This operation was repeated three times to improve BTA loading efficiency. At the same time, in order to remove excess BTA not loaded on Hap−CD, the suspension was washed three times with deionized water and centrifuged at 5000 rpm/min. Finally, the prepared product was dried in a vacuum oven at 50 °C for 12 h.

#### 3.2.5. Preparation of BTA−HAp−CD/EP Coating

First, the N80 steel sheet was processed into a rectangular parallelepiped shape of 1 cm × 1 cm × 3 mm. Then, all the finished metal steel sheet surfaces were polished with 400 mesh, 800 mesh, and 1200 mesh sandpaper in turn, and then cleaned and degreased with anhydrous ethanol and acetone, respectively, and dried in a vacuum oven. Thereafter, 0.1 g of the BTA−HAp−CD material was added into 20 g epoxy resin for magnetic stirring to ensure uniform dispersion of nanomaterials in epoxy resin, and then a 2.9 g curing agent was added for another stirring. The mixture was degassed for 30 min. Finally, the composite coating was applied to the surface of the pretreated N80 metal steel sheet and cured at room temperature for 24 h, followed by 4 h in a vacuum oven at 80 °C. The coating sample obtained by this method was named BTA−HAp−CD/EP. For the parallel comparison experiment, pure epoxy resin coating (pure EP), the HAp/EP coating, HAp−CD/EP coating, and BTA−HAp−CD/EP coating without any nano−filler were prepared by the same method.

### 3.3. Characterization Test

Fourier Transform infrared spectroscopy (FT−IR) detection was performed via a WQF−520 machine manufactured by Beijing Rayleigh Analytical Instrument Company (Beijing, China). The lattice parameter test (XRD) utilized the X ‘Pert Pro MPD produced by Panaco, the Netherlands. The test used a Cu target and a Kα radiation source, and the tube current and tube voltage were 40 mA and 40kV, respectively, the wavelength was 1.54 A, the scanning range of the diffractometer was 5–70°, and the scanning speed was 5°/min. The ESCALAB 250 X−ray photoelectron spectrometer manufactured by Waltham Corporation (Boston, USA) was used for elemental analysis (XPS). A scanning electron microscope (JSM−7500F) produced by Japan Electronics Company (Tokyo, Japan) was used to observe the microstructure of the samples. The electrochemical test (EIS) used the CHI604D electrochemical workstation produced by the Shanghai Chenhua Company (Shanghai, China). The electrochemical test system was a classic three−electrode system, which consists of a working electrode, platinum electrode as an auxiliary electrode, and a saturated calomel (SCE) electrode as the reference electrode. Corrosion inhibitor release detection (UV−vis) was performed using a UV−5800 ultraviolet spectrophotometer produced by Yuanxi Corporation (Shanghai, China).

## 4. Conclusions

In general, a composite epoxy coating based on HAp with a PH−response function was successfully prepared. Firstly, the synthesized composite material was characterized by FTIR, XRD, XPS, and SEM and compared with each monomer material, proving the successful preparation of BTA−HAp−CD. Secondly, from the results of the sustained−release agent release experiment, it can be determined that the positive impact of composite materials in the corrosion process is better than that of other materials, which can also be demonstrated from the electrochemical results. Moreover, the optical images of the salt spray test clearly showed the excellent anti−corrosion properties of the prepared epoxy coating. The enhancement of HAp on the physical barrier properties of the coating and the protective adsorption layer formed on the metal surface after the release of BTA conferred the coating a good pH response and self−healing properties. In short, this paper presented a new intelligent coating design scheme that showed great application prospects.

## Data Availability

The data that has been used is confidential.

## Supplementary Materials

The following supporting information can be downloaded at: https://www.mdpi.com/article/10.3390/molecules28176223/s1, Table S1: Electrochemical fitting parameters of coatings; Table S2: Z_f=0.01Hz_ of scratch test; Figure S1: Experiments on dispersibility of different materials in deionized water (a–c) and ethanol (d–f). From left to right, the bottles are HAp, F-HAp, HAp-CD and BTA-HAp-CD; Figure S2: TGA testing of composite coatings.

## Sample Availability

Samples of the HAp, F−HAp, HAp−CD, and BTA−HAp−CD are available from the authors.
