# Supplementary material for: A High−Performance Anti−Corrosive Epoxy Coating Based on Ultra−Thin Hydroxyapatite Nanosheets with pH−Responsive Functions
Source: Molecules. 2023 Aug 24;28(17):6223. doi: 10.3390/molecules28176223 (PMC10488751; doi:10.3390/molecules28176223)
Supplement: Supplementary file 1 [file molecules-28-06223-s001.zip › molecules-2496340-supplementary.pdf]

A High−Performance Anti−Corrosive Epoxy Coating Based on Ultra−Thin Hydroxyapatite Nanosheets with pH−Responsive Functions

Chun Feng [1,2], Lijuan Zhu [1,2,*], Legang Chen [3], Xuezhi Hui [4], Jinling Liu [5], Lei He [1,2], Xiaofeng Bai [1,2] and Zongxue Yu [3,*]

[1] Tubular Goods Research Institute, China National Petroleum Corporation, Xi’an 710077, China
[2] State Key Laboratory for Performance and Structure Safety of Petroleum Tubular Goods and Equipment Materials, Xi’an 710077, China
[3] School of Chemistry and Chemical Engineering, Southwest Petroleum University, Chengdu 610500, China
[4] Petrochina Changqing Oilfield Company, China National Petroleum Corporation, Xi’an 710021, China
[5] Bureau of Geophysical Prospecting Inc., China National Petroleum Corporation, Zhuozhou 072751, China
* Correspondence: zhulijuan1986@cnpc.com.cn (L.Z.); 201131010005@swpu.edu.cn (Z.Y.)

Table. S1 Electrochemical fitting parameters of coatings

Sample	Time (Day)	Open Potential (V)	log f_b (Hz)	log $Z_{f=0.01Hz}$ (Ω cm^2)	R_c (Ω cm^2)	R_{ct} (Ω cm^2)
Pure EP	1	-0.13	0.33	8.03	1.03E8	
	3	-0.14	0.46	7.86	6.97E7	
	7	-0.26	1.25	7.11	1.29E7	
	14	-0.34	1.41	6.83	8.48E6	
	20	-0.57	3.40	5.61	3.65E5	1.12E5
	30	-0.68	4.10	4.81	5.78E4	2.17E4
HAp/EP	1	-0.10	-0.08	8.39	2.30E8	
	3	-0.12	0.34	7.98	9.38E7	
	7	-0.21	0.92	7.43	2.73E7	
	14	-0.27	2.49	6.37	2.20E6	8.01E5
	20	-0.52	3.88	5.31	2.01E5	3.40E4
	30	-0.65	4.07	4.87	6.60E4	2.46E4
F-HAP/EP	1	-0.016	-0.20	8.50	2.94E8	
	3	-0.064	0.21	8.13	1.32E8	
	7	-0.18	0.69	7.66	4.51E7	
	14	-0.24	1.17	7.19	1.52E7	
	20	-0.48	3.03	6.08	1.12E6	2.66E5
	30	-0.62	3.07	5.62	3.80E5	2.29E5
HAp-CD/EP	1	0.079	-0.33	8.61	3.77E8	
	3	-0.023	0.10	8.18	1.51E8	
	7	-0.14	0.47	7.82	6.33E7	
	14	-0.22	0.77	7.57	3.50E7	
	20	-0.39	1.53	6.83	6.78E6	6.12E6
	30	-0.463	1.97	6.44	2.74E6	2.62E6
BTA-HAp-CD/EP	1	0.231	-0.41	8.74	5.89E8	
	3	0.028	-0.22	8.51	2.64E8	
	7	-0.12	0.38	7.96	8.71E7	
	14	-0.16	0.50	7.76	5.75E7	
	20	-0.19	0.56	7.86	6.84E7	6.72E7
	30	-0.20	0.63	7.80	6.10E7	6.01E7

Table. S2 $Z_{f=0.01Hz}$ of scratch test.

Sample	Time（h）	log $Z_{f=0.01Hz}$ (Ω cm^2)
Pure EP	2	3.71
	24	3.66
	72	3.57
	168	3.22
HAp/EP	2	3.94
	24	3.84
	72	3.71
	168	3.60
F-HAp/EP	2	4.45
	24	4.05
	72	3.76
	168	3.65
HAp-CD/EP	2	4.46
	24	4.30
	72	3.95
	168	3.84
BTA-HAp-CD/EP	2	4.55
	24	4.64
	72	5.38
	168	4.77

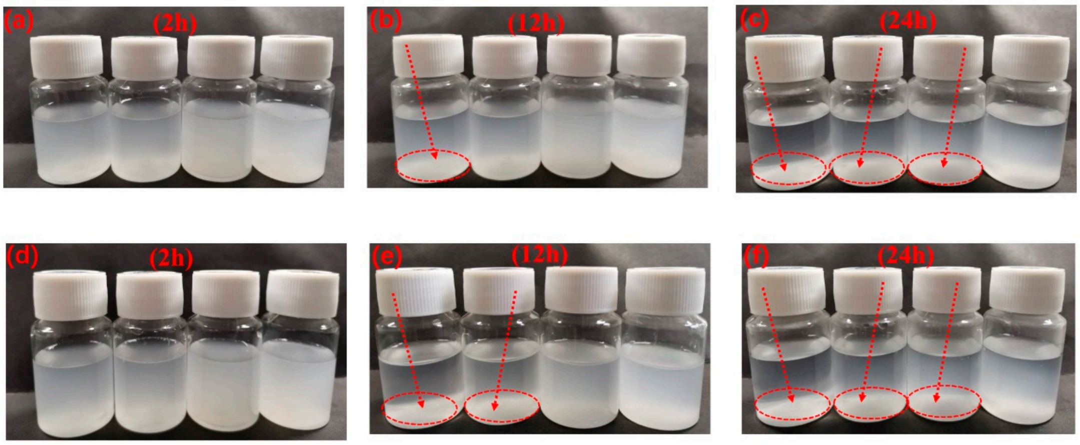

Fig. S1 Experiments on dispersibility of different materials in deionized water (a-c) and ethanol (d-f). From left to right, the bottles are HAp, F-HAp, HAp-CD and BTA-HAp-CD.

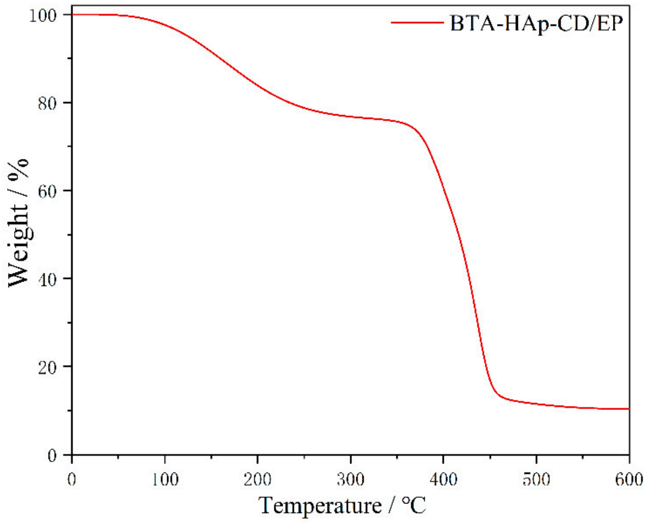

Fig. S2 TGA testing of composite coatings.
